# A Rare Case Report of Dental and Craniofacial Manifestations of Nemaline Myopathy

**DOI:** 10.7759/cureus.49091

**Published:** 2023-11-20

**Authors:** Sheetal Badnaware, Pooja Gupta

**Affiliations:** 1 Department of Pedodontics and Preventive Dentistry, Faculty of Dental Sciences, Institute of Medical Sciences, Banaras Hindu University, Varanasi, IND

**Keywords:** gonial angle, nebulin gene, anterior open bite, triple furrow sign, nemaline myopathy

## Abstract

Any congenital muscular disorder can cause severe dental and craniofacial deformity. The clinicians must understand the aetiology of this muscular disorder to plan the treatment for this condition. Currently, there is limited data available in the literature on the dental manifestation of nemaline myopathy. Nemaline myopathy is a type of rare congenital muscular disorder characterized by severe dental and craniofacial deformity. This case report describes the dental and craniofacial manifestations of such diseases in an eight-year-old child who visited the unit of pediatric and preventive dentistry with a chief complaint of irregular placement of teeth and inability to close the mouth.

## Introduction

Nemaline myopathy is a congenital non-dystrophic muscular disorder affecting early infancy or childhood with an incidence of 2 in 100,000 live births [1]. The prevalence rate of nemaline myopathy is 1:22,480 and in a recent systematic review, a total number of 55 cases have been reported from 101 paediatric patients from 23 countries [2,3]. The exact data still need further investigation. The most common complications of nemaline myopathy are respiratory insufficiency, cardiopulmonary arrest, infection and sepsis affecting the quality of life of individuals. It was first described by Shy et al. in 1963 describing the meaning of Nema means threads, which describes the thread-like bodies that run longitudinally to muscle fibres [4]. The nemaline bodies or rods are distributed in muscle fibre randomly and visible after staining with the Gomori trichrome method. The size of these rods under an electron microscope is 1-7um in length and 0.3-2 um in width [4]. Diagnosis of these myopathy is made histologically by identifying the presence of nemaline bodies in muscle biopsy which is present in the sarcolemmal membrane of muscle fibre. Currently, at least 12 genes have been related to this disease with which Nebulin (NEB) is the most common gene associated with nemaline myopathy [5]. Nebulin is one of the several proteins that interact to generate the mechanical force for muscle contraction. Most of the congenital myopathy are heterogeneous group of neuromuscular disorders causing weaking of proximal facial muscles, neck flexor, respiratory and bulbar involvement. According to parental history, since birth child has been facing respiratory difficulty and loss of motor function like gait disturbances, walking and difficulty in climbing and getting up from squatting position. A child had delayed motor milestones. There are seven genotypical types of nemaline myopathy and according to the European Neuromuscular Center International Consortium classification, there are six types based on decreasing severity. The severe congenital form, intermediate congenital form, the typical congenital type, childhood-onset, adult-onset and other forms of nemaline myopathy. Based on genetic testing, the molecular aetiology present case is type 2 nemaline myopathy which has the NEB gene [1]. Patients with nemaline myopathy have a wide range of clinical and craniofacial manifestations including elongated face, high-arched palate and facial muscle hypotonia which is rarely discussed in the literature. In the Indian population, among 100 cases of congenital myopathy over a period of 20 years, only four cases of nemaline myopathy were recorded in south India and in North India, four cases of nemaline myopathy were recorded in 15 congenital myopathy cases [6]. The dental and craniofacial manifestations of nemaline myopathy are under-reported in India. This case report describes the dental and craniofacial manifestations of nemaline myopathy in an eight-year-old male child.

## Case presentation

**Figure 1:**
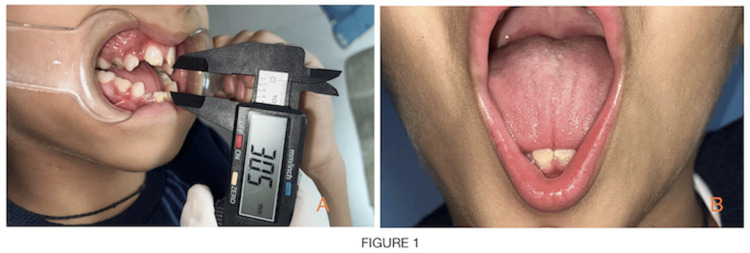
Intraoral view showing increased anterior open bite of more than 30mm (A) and triple furrow sign on the tongue (B).

**Figure 2:**
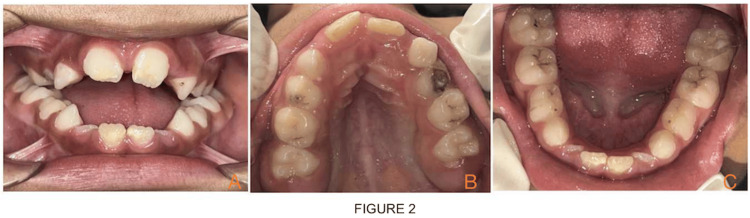
Intraoral view showing hypoplastic incisors teeth (A) and maxillary occlusal view showing deep narrow palatal and constricted maxillary arch with enlargement of palatal mucosa (B) and mandibular occlusal view showing inward inclination of posterior teeth (C).

**Figure 3:**
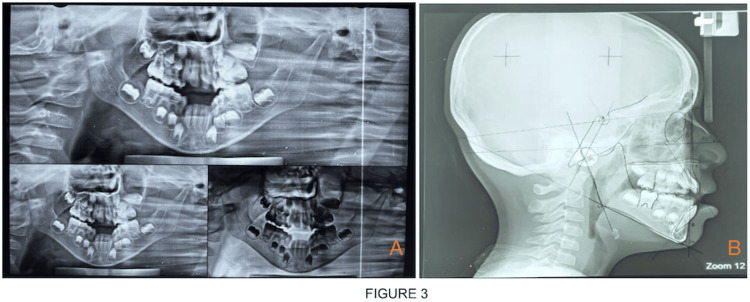
(A) Panoramic radiograph. (B) Lateral cephalometric tracing.

**Figure 4:**
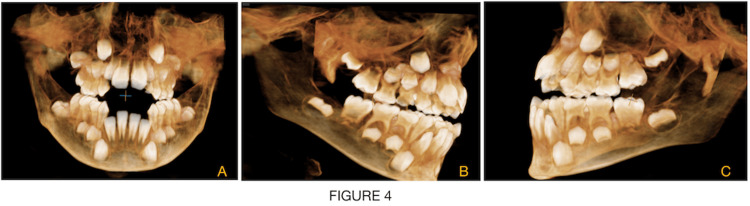
CBCT (cone beam computed tomography) scan confirms the Nolla stages of teeth development. 4A - front view, 4B - right lateral view and 4C - left lateral view

An eight-year-old male child was brought to the unit of pediatric and preventive dentistry with a chief complaint of irregular placement of teeth and inability to close the mouth. The child had been diagnosed with nemaline myopathy on genetic testing. According to parental history, from the age of two years, a child was having gait disturbances, muscle weakness, difficulty in getting up from squatting position and difficulty in climbing stairs and jumping. Respiratory problems have been common in this kind of patient since childhood. A child had delayed motor milestones. A child started sitting around the age of nine months and was able to stand at 19 months. The marriage of parents was consanguineous. On physical examination, there is significant atrophy of the proximal muscle, face and masticatory muscle and gait is wadding. On extraoral examination, a child had a dolichocephalic pattern with a bilaterally symmetrical face, increased lower facial height, increased gonial angle, drooping of the corner of the mouth and midface retrusion or hypoplasia. The characteristic of a myopathic face is seen on clinical examination, i.e., elongated and expressionless face, high arched palate (V-shaped deep palate with constriction of maxilla) and mouth tent-shaped. A child's mouth remains open and is unable to close passively. Face length is elongated craniocaudally. The side profile of the child is straight with posterior divergence. On intraoral examination, the characteristic finding, in this case report noted, is an increased open bite of more than 30mm and a triple furrow sign on the tongue with the presence of atrophy (Figures 1A, 1B). Hypoplastic permanent incisors and mouth breathing habits were also noted (Figure 2A). The upper arch is a deep narrow palate and marked enlargement of palatal mucosa was seen palatally with mild posterior crossbite bilaterally and in the mandible, inward inclination of posterior teeth was seen (Figures 2B, 2C). There is a class 1 molar relation on both sides with an inability to close properly due to retrognathia of the mandible all deciduous molars were carious and oral hygiene was poor. On the orthopantomographic (OPG) examination (Figure 3A), there is a presence of all permanent tooth buds, including the third molar. On the CBCT (cone beam computed tomography) scan (Figures 4A, 4B, 4C), Nolla stage 7 confirms with 11, 21, 31, 41, 12, 22, 16, 26, 36 and 46 permanent teeth. Nolla stage 4 confirms with 17, 37, 47 and 27 permanent teeth and Nolla stage 5 confirms with 13, 14, 15, 23, 24, 25, 32 and 42 permanent teeth. Nolla stage 6 confirms with 33 and 43 teeth. On lateral cephalometric tracing, there is an increase in gonial angle, i.e., 138 degrees and an increase in the lower anterior facial (Table 1; Figure 3B). The length of the maxilla and mandible is also reduced. Frankfurt mandibular angle is increased indicating an increase in the lower anterior facial height. Management of orthodontic problems was discussed with the parents.

**Table 1:** Cephalometric measurement of patients N-S-Ar: Nasion-Sella-Articulare, SNA: Sella-Nasion-A, SNB: Sella-Nasion-B, ANB: A-point-Nasion-B-point, SN length: Sella-Nasion length, SN-MP: Sella-Nasion to Menton-Pogonion, FMA: Frankfort mandibular angle, IMPA: Incisor mandibular plane angle

Measurement	Normal value	Measured value
Saddle angle (N-S-Ar)	123^0^	121^0^
SNA	82^0^	81^0^
SNB	80^0^	79^0^
ANB	2^0^	2^0^
Articular Angle	143^0^	142^0^
Gonial Angle	123^0^	138^0^
Axis	66^0^	67^0^
Inter-Incisal Angle	123^0^	119^0^
SN Length	65mm	50mm
Maxillary Length	44mm	39.5mm
Mandibular Length	69mm	52mm
SN-MP	32^0^-35^0^	41^0^
FMA	25^0^	35^0^
IMPA	87^0^	91^0^

Laboratory investigations

All the blood liver and kidney profiles were within the normal range. Whole exome sequencing for these children was revealed on molecular testing using next-generation sequence (NGS) heterozygous c.2784de IT (p.Asp929llefs*28) likely pathogenic variant in NEB gene was observed in these children and heterozygous c.21522+3A>G variant of uncertain significance in NEB gene was observed in this individual. The child was diagnosed with an autosomal recessive pattern.

## Discussion

This case report describes how the congenital muscular disorder can affect the growth of the craniofacial complex causing severe malocclusion. The prevalence rate in children is higher than the prevalence in adults because children with myopathy may not survive after particular age groups. The prevalence rate of congenital myopathy for children is 2.76 in 100000 in pediatric patients and for nemaline myopathy, the overall prevalence rate was 0.22 per 100,000 [7]. Positive family history and changes in the gene pattern mutations may be the important risk factors for the congenital type of myopathy like nemaline myopathy with autosomal recessive pattern with the most common inheritance. A total number of 10 types of gene mutations have been identified for this condition. NEB-1 and ACTA 1 are the most common genes responsible for these conditions.The autosomal recessive form is more common in childhood and the autosomal dominant form is common in adulthood. For the acquired type of myopathy, the risk factor is autoimmune diseases (polymyositis, dermatomyositis, etc.), drug-induced myopathy (glucocorticoids, D-penicillamine or lipid-lowering agents, etc.), viral infections (mumps, Coxsackie, influenza virus, etc.), endocrine disorder (hypothyroidism or hyperthyroidism, etc.). Various causative factor has been noted in the literature for malocclusion including genetic disorder, hereditary conditions, environmental elements affecting the fetus's injuries, premature tooth loss, dysfunctions and parafunction of stomatognathic apparatus, and other diseases [8]. Various researchers have noted the impact of muscular disorder involvement in the growth of the craniofacial complex causing facial dysmorphogenesis and functional malocclusion [9,10]. There are few studies regarding this noted in the literature [11,12]. Anterior open bites and long faces are the most common dental findings noted in most muscular disorders which is also seen in our case report [1]. Similar to our case report, Deans et al. [1] and Xue et al. [13] describe the anterior open bite and long faces are characteristic features of nemaline myopathy. Currently, there is no definitive treatment for such congenital myopathy. Treatment planning needs to take into account the severity and progression of the disease. Biopsy and genetic molecular investigation are critical for the management of congenital myopathy. It comprises an interdisciplinary team of paediatric dentists, orthodontists, oral surgeons, craniofacial team neuromuscular surgeons, etc. The severe malocclusion requiring extensive orthodontic surgery should be deferred due to difficulty in intubation and tracheostomy for anaesthetics. Succinylcholine should be avoided in such cases. The anaesthetic risk and surgical stability of the procedure should be considered while planning any surgical procedure for such cases [13]. The concomitant theory said that the reason for the anterior open bite and long faces is caused by the mandible growing downwards and forwards resulting in the increased lower facial height, marked large tongue size and low tongue position. Dystrophy of facial muscle causes the mandible clockwise rotation due to gravity or the absence of suprahyoid muscle pull. This affects the transverse position of the teeth, reducing palatal width and causing posterior crossbite in such cases. Mandibular rotation in a clockwise direction also increases the angle between the mandibular plane and the palatal plane also increases the gonial angle and contributes to the mouth-breathing habit in this case report. A triple furrow sign with the presence of tongue atrophy was also noted in the case report similar to another author [5]. Quereshy FA et al. [2] in their case report mentioned severe anterior open bite, high-arched palate, and mandibular retrognathia are characteristic oral features of nemaline myopathy and Yoo et al. [14] also mentioned anterior open bite and mouth breathing habit and drooling in his case report. The current literature available on craniofacial management of nemaline myopathy is very limited. The first case report published on orthodontic surgery for nemaline myopathy in mixed dentition was by Quereshy FA et al. [2] in the year 2020. Oral care and general care for nemaline myopathy should follow the guidelines reviewed in the consensus statement of the standard of care for congenital myopathy as there is a paucity of literature regarding standard treatment protocol for muscle disorder patients [15]. Due to the multisystem involvement in nemaline myopathy patients require a multidisciplinary approach from various healthcare professionals to improve the quality of healthcare life. The anticipatory guidance for these patients should start from the first tooth erupts in the oral cavity. During the first year of life, feeding difficulty is the most commonly seen problem. It should be monitored with increased age otherwise alternative gastric feeding tubes should be considered. Feeding issues can impair the overall growth and development of patients. Monitoring of growth with respect to height and weight should be done at least every three months. Orofacial problems such as malocclusion, high arched palate and lower facial muscle contractures are seen as major problems in this disease. Various behaviour management techniques such as desensitizing techniques should be used for proper care of the oral cavity. Evaluation of high-arched palate and malocclusion should be at the age of six to eight years. Surgical planning for severe malocclusion should not be considered due to the high severe risk of complications such as difficulty in intubation and anaesthesia. The milder form of malocclusion should be treated after consultation with physicians to avoid respiratory difficulty for patients. Difficulty of speech problems is due to muscle hypotonia or abnormal anatomy. Referral speech pathologists should be considered at the earliest. Oral motor therapy should be considered useful to train patients with nemaline myopathy like lip exercise (oral screen) to prevent mouth breathing habitat. Excessive drooling of saliva is a common problem seen in muscle disorder patients and should be treated with pharmacologic agents such as anticholinergic drugs. Recommendations of this guideline include referral to a neurologist, pulmonologist, orthopedician, physiotherapist, occupational therapist and speech therapist should be done at the earliest to reduce morbidity. No specific pharmacologic treatment is available. Monitoring of respiratory function and spine should be done during every follow-up visit of patients. The follow-up care of patients should include the examination of cardiac status due to the risk of cardiac disease. The success of treatment for these patients requires coordinated oral and general care. The child has been recalled every three to four months for follow-up care. Only preventive type of treatment is given to the child including oral hygiene instructions and fluoride application. After consultation with the orthodontic team, extensive orthodontic surgery is delayed at an early age due to lower facial muscle weakness. Increase effort has been asked the child to produce a lip seal. The oral screen has not been given for this child due to respiratory difficulty. During the management of nemaline myopathy difficulty in intubation and complications from anaesthesia should be considered [15]. According to Bagnall, there is no current specific intervention to overcome this structural facial progression [16]. This case report describes the orofacial features of nemaline myopathy.

## Conclusions

The present findings of the case report suggest that patients with nemaline myopathy exhibit numerous changes in the dentofacial complex with increases in the prevalence of malocclusion. In congenital muscular disorders due to the lower facial muscle weakness and respiratory difficulty the early orthodontic intervention in such cases is not possible. To intervene in such cases at the early ages more research is needed.
